# Hepatitis C Virus Attenuates Mitochondrial Lipid β-Oxidation by Downregulating Mitochondrial Trifunctional-Protein Expression

**DOI:** 10.1128/JVI.01653-14

**Published:** 2015-02-11

**Authors:** Yutaka Amako, Tsubasa Munakata, Michinori Kohara, Aleem Siddiqui, Chris Peers, Mark Harris

**Affiliations:** a School of Molecular and Cellular Biology, Faculty of Biological Sciences, University of Leeds, Leeds, United Kingdom; b Department of Microbiology and Cell Biology, Tokyo Metropolitan Institute of Medical Science, Tokyo, Japan; c Faculty of Medicine and Health, University of Leeds, Leeds, United Kingdom; d Department of Medicine, Division of Infectious Diseases, University of California, San Diego, California, USA

## Abstract

The course of hepatitis C virus (HCV) infection and disease progression involves alterations in lipid metabolism, leading to symptoms such as hypocholesterolemia and steatosis. Steatosis can be induced by multiple mechanisms, including increases in lipid biosynthesis and uptake, impaired lipoprotein secretion, and/or attenuation of lipid β-oxidation. However, little is known about the effects of HCV on lipid β-oxidation. A previous proteomics study revealed that HCV interacted with both the α- and β-subunits of the mitochondrial trifunctional protein (MTP), an enzyme complex which catalyzes the last 3 steps of mitochondrial lipid β-oxidation for cellular energy production. Here we show that in HCV-infected Huh7.5 cells, lipid β-oxidation was significantly attenuated. Consistently with this, MTP protein and mRNA levels were suppressed by HCV infection. A loss-of-function study showed that MTP depletion rendered cells less responsive to alpha interferon (IFN-α) treatment by impairing IFN-stimulated gene expression. These aspects of host-virus interaction explain how HCV alters host energy homeostasis and how it may also contribute to the establishment of persistent infection in the liver.

**IMPORTANCE** HCV infection triggers metabolic alterations, which lead to significant disease outcomes, such as fatty liver (steatosis). This study revealed that HCV impairs mitochondrial lipid β-oxidation, which results in low lipid combustion. On the other hand, the HCV-induced defects in metabolic status played an important role in the control of the type I interferon system. Under the conditions of impaired lipid β-oxidation, host cells were less responsive to the ability of exogenously added IFN-α to suppress HCV replication. This suggests that interference with lipid β-oxidation may assist the virus in the establishment of a long-term, persistent infection. Further understanding of this aspect of virus-host interaction may lead to improvements in the current standard therapy.

## INTRODUCTION

Hepatitis C virus (HCV) is a member of genus Hepacivirus in the family Flaviviridae. Its genome consists of up to 9,600 bases of single-stranded RNA with positive polarity. Genomic RNA contains a single open reading frame encoding a polyprotein (∼3,000 amino acids), which is processed into 10 mature proteins by both cellular and viral proteases. The 10 mature proteins consist of three structural proteins (core, E1, and E2), p7, and the nonstructural (NS) proteins NS2, NS3, NS4A, NS4B, NS5A, and NS5B (1). HCV is a global health problem; it chronically infects ∼170 million people and is a leading cause of liver transplantation. Because of the lack of a prophylactic vaccine and limitation of effective therapeutic options, persistent HCV infection leads patients to chronic hepatitis, fatty liver (steatosis), cirrhosis, and liver cancer (2, 3).

Until recently, the standard of care was 24 to 48 weeks of therapy with pegylated interferon (IFN) and ribavirin. Success rates of this therapy differed greatly, from 6% to 84%, depending on the patient's situation (viral load and genotype, severity of liver disease, etc.). Direct-acting antivirals, which include viral protease (NS3), polymerase (NS5B), and NS5A inhibitors, are now approved for clinical treatment (4). Although a combination of these inhibitors will soon replace current standard therapy, the potential for the emergence of resistant virus is high, due to the high rate of turnover of virus and the high error rate of the NS5B polymerase (5).

A body of evidence shows that the course of disease progression involves metabolic alteration of lipid biogenesis and its homeostasis in the liver (6). Furthermore, viral replication and egress have been reported to correlate with very-low-density lipoprotein (VLDL) biogenesis and the secretion pathway (7). It has been shown that viral genomic replication takes place on altered endoplasmic reticulum and is tightly associated with cytosolic lipid droplets (8). The following stages of viral maturation/release have been reported to be coupled with VLDL secretion, so that infectious viral particles appear to associate with certain lipoproteins to form lipo-viro-particles (LVPs) (7, 9–12). In this regard, HCV hijacks the VLDL biogenesis/secretion pathway (13). Since the liver is the most important organ for lipid transportation and storage, understanding why the entire viral life cycle is so tightly associated with lipid biogenesis and transport will be key to an understanding of the mechanisms of viral invasion and pathogenesis.

While HCV utilizes VLDL biogenesis for its dissemination, viral infection causes fatty liver (steatosis), which potentially worsens chronic inflammation (steatohepatitis), most likely due to reactive oxygen species (ROS) in the liver (14, 15). Furthermore, fatty liver is a common risk factor for the development of insulin resistance (16). Chronic HCV infection can thus be regarded as a metabolic syndrome. Although many studies have investigated the upregulation of lipogenesis by HCV (17, 18), little is known about the effect of virus infection on mitochondrial lipid β-oxidation, apart from one study suggesting that HCV might impair this process *in vivo* (19). Since mitochondrial lipid β-oxidation yields a greater amount of ATP than carbohydrate or protein consumption, host energy metabolism may be unbalanced when it is negatively impacted by viral infection. Indeed, recent studies suggest that HCV infection triggers a shift in the energy expenditure profile in terms of glucose consumption and production (20–22).

Our previous characterization of the protein interactome of the HCV nonstructural protein NS5A suggested that it interacts with both the α- and β-subunits of mitochondrial trifunctional protein (MTP) (23). The MTP complex, which is an octamer of 4 α-subunits and 4 β-subunits, has three enzymatic activities, which catalyze the last stages in mitochondrial lipid β-oxidation: 3-hydroxyl–coenzyme A (CoA) dehydrogenase, 3-ketoacyl–CoA thiolase, and enoyl-CoA hydratase. Interestingly, MTPα heterozygous (MTPα^+/–^) transgenic mice have been shown to develop fatty liver and insulin resistance (24). In this study, we aim to dissect the molecular mechanism underpinning the effect of viral infection on lipid β-oxidation and the possible impact of this on the innate immune response to HCV.

## MATERIALS AND METHODS

### Plasmids.

pJFH1 was provided by Takaji Wakita (National Institute of Infectious Disease, Japan) and described previously (25). The pJc1 construct was a gift from R. Bartenschlager (University of Heidelberg) and used to construct the pJc1-p7NLuc2A reporter virus construct (26). pJc1-p7NLuc2A and pJc1-p7NLuc2A/GND were constructed by conventional molecular cloning methods, and nucleotide sequences are available upon request. pISRE-Luc was purchased from Agilent Technologies. pRL-TK was from Promega.

### Cell culture and virus infection.

Huh7.5 cells were a gift from C. Rice (Rockefeller University). The 293FT cell line is from Life Technologies and was used for lentiviral packaging. Huh7.5 cells were maintained in high-glucose Dulbecco's modified Eagle medium supplemented with 10% fetal bovine serum, 100 U/ml penicillin, 100 μg/ml streptomycin, 1 mM nonessential amino acids, and 2 mM GlutaMAX (Life Technologies). To generate viruses, vectors were linearized by XbaI digestion followed by mung bean nuclease treatment to blunt the digested termini and used as template DNAs for T7 promoter-driven *in vitro* transcription using the T7 RiboMAX large-scale RNA production system (Promega, Madison, MA). RNA transfection was performed as described previously (25). Cultured virus titers were determined as described in reference 28.

### Lentiviral vectors for expressing shRNA.

pCS-RfA-EG and a set of packaging plasmid vectors, including pCMV-VSV-G-RSV-Rev and pCAG-HIVgp, were provided by Hiroyuki Miyoshi (Riken BioResorce Center, Japan). To construct short-hairpin RNAs (shRNAs) expressing lentiviral vectors, a pair of 65-mer oligonucleotides were inserted into BglII and XbaI sites of pENTR4-H1. Oligonucleotide sequences for constructing luciferase mRNA-specific shRNA (shLuc), short-hairpin green fluorescent protein (shGFP), shLacZ, shMTPα, and shMTPβ are available upon request. The shRNA-encoding insert was confirmed by DNA sequencing. The shRNA expression cassette of pENTR4 was inserted into pCS-RfA-EG by Gateway LR Clonase II (Invitrogen)-directed DNA recombination as per the manufacturer's recommendations. Lentiviral particles were obtained as cultured supernatants from packaging transfections performed using Fugene 6 (Promega), as per the manufacturer's recommendations.

### NanoLuc reporter virus and replication assay.

T7 RNA transcription and electroporation for pJc1-p7NLuc2A were performed as described above. Reporter virus titers in cultured supernatants were determined by focus-forming unit (FFU) assay. Naive Huh7.5 cells were infected at a defined multiplicity of infection (MOI) of 0.05 to initiate virus culture. The nanoluciferase (NanoLuc) assay was performed using the Nano-Glo luciferase assay system according to the manufacturer's instructions.

### Fatty acid β-oxidation assay.

Huh7.5 cells were infected with JFH-1 virus at an MOI of 0.3. [^3^H]palmitic acid was conjugated to fatty-acid-free bovine serum albumin (BSA). HCV-infected cells were incubated with Krebs's buffer containing [^3^H]palmitic acid-BSA for 2 h at 37°C. The amount of released ^3^H_2_O was measured using an LSC-6100 scintillation counter (Aloka) as described previously (29). Etomoxir and l-carnitine were used at 20 μM and 1 mM, respectively.

### Real-time PCR.

Quantification of HCV RNA and GAPDH (glyceraldehyde-3-phosphate dehydrogenase) mRNA was performed as described previously (30). Primer pairs for quantification of MTPs, β-actin, and proliferator-activated receptor γ coactivator 1α (PGC-1α) mRNA are available upon request. Real-time PCR was performed using Superscript III (Life Technologies) for first-strand synthesis and QuantiFast SYBR green (Qiagen) according to the manufacturers' instructions. Mitochondrial DNA (mtDNA) quantitation was performed as described previously (31).

### Interferon promoter/reporter assay.

Plasmids pISRE-Luc and pRL-TK (20:1, wt/wt, ratio) were transfected into Huh7.5 cells on a 12-well plate with Fugene 6 transfection reagent. Transfected cells were split into a 96-well plate at 24 h posttransfection. Cells were further incubated for 48 h, and during the last 5 h, they were exposed to IFN-α at the concentrations indicated in Fig. 6. Measurement of luciferase activity was performed using the dual-luciferase reporter assay system for pISRE-Luc and pRL-TK.

### Protein assay.

Western blotting was performed using the Odyssey Sa infrared imaging system (LI-COR). Detected protein band intensities were analyzed by Image Studio software (LI-COR).

### Antibodies and reagents.

Human IFN-α was purchased from Mochida Seiyaku (Japan). Commercially obtained antibodies are anti-MTPα and -MTPβ (Santa Cruz Biotechnology), anti-protein disulfide isomerase (PDI), and β-actin (Sigma-Aldrich).

## RESULTS

### HCV infection attenuates lipid β-oxidation.

**FIG 1:**
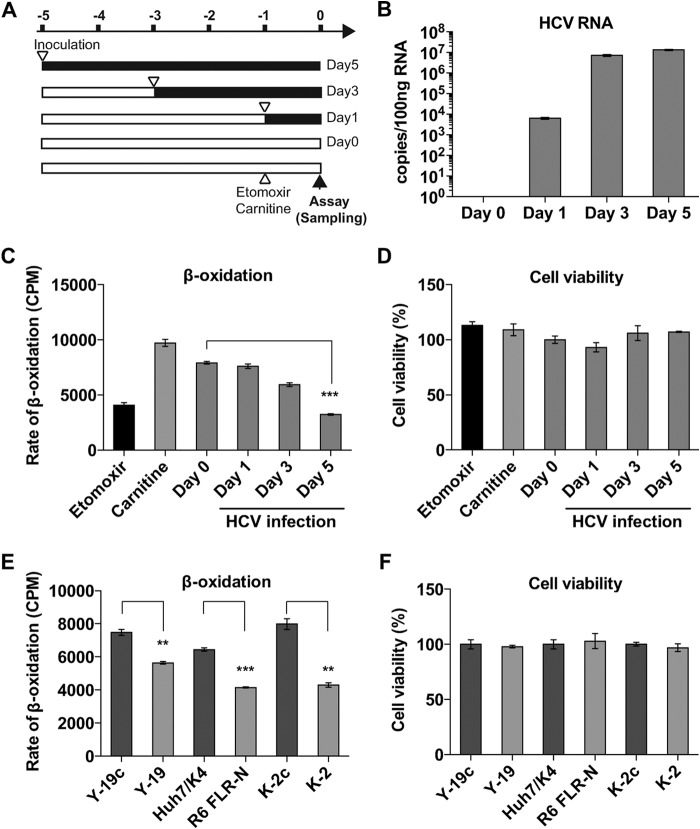
HCV infection attenuates lipid β-oxidation. (A) Schematic of experiment. Huh7.5 cells were plated at 1.6 × 10^4^ cells per well in a 24-well plate and then infected with HCV JFH-1 at different times (MOI of 0.3 FFU/cell), so that the lipid β-oxidation assay could be performed for all samples simultaneously. (B) Cellular HCV RNA was measured by real-time quantitative RT-PCR. (C) Huh7.5 cells were fed with [^3^H]palmitate, and ^3^H_2_O was assayed as a measure of lipid β-oxidation. The rate of lipid β-oxidation decreased in a time-dependent manner. Huh7.5 cells were also treated with etomoxir (20 mM) or l-carnitine (1 mM) as a known inhibitor or enhancer of mitochondrial lipid β-oxidation, respectively. (D) Cell viabilities were unaffected and confirmed by using a CCK-8 assay. (E) The same lipid β-oxidation assay was performed for HCV replicon cells. Y-19 is a stable genotype 2a JFH-1 SGR-harboring cell line, and Y-19c is the corresponding IFN-cured cell line. R6 FLR-N is a genotype 1b SGR-harboring cell line, and Huh7/K4 is the corresponding cured cell line. K-2 is a JFH-1 full-length replicon (FLR)-harboring cell line, and K-2c is the corresponding cured cell line. (F) Replicon RNA replications did not affect cell viabilities. Column error bars represent the standard errors of the means (SEM). Unpaired Student *t* tests were performed in order to calculate statistical significance between indicated columns. Asterisks indicate *P* values as follows: *, <0.05; **, <0.001; and ***, <0.0001.

It was previously shown that HCV infection led to a reduction in ATP production in tissue culture (32). It has also been shown that HCV proteins interact with mitochondria to negatively impact mitochondrial functions and/or modulate innate immune signaling cascades, which utilize mitochondrial membranes as a platform (14, 15, 33, 34). We previously showed that NS5A interacts with MTPα and MTPβ (also known as 3-hydroxyl-CoA dehydrogenase/3-ketoacyl-CoA thiolase/enoyl-CoA hydratase, alpha and beta subunits [HADHA and HADHB], respectively). MTPβ was also proposed to be involved in HCV-induced metabolic changes within infected cells following another proteomic analysis (18). These data suggest that the major mitochondrial lipid β-oxidation pathway might be affected by HCV infection, which might in turn cause impaired ATP production and alter the balance of energy homeostasis. To assess this possibility, we performed a lipid β-oxidation assay using [^3^H]palmitate as a tracer. The results clearly indicated that HCV infection significantly attenuated lipid β-oxidation (Fig. 1C) in a time-dependent manner that correlated with the progress of HCV infection, with inhibition reaching 59.3% at day 5 (Fig. 1C). Treatment of uninfected Huh7 cells with either etomoxir or l-carnitine (as a known inhibitor or enhancer of mitochondrial lipid β-oxidation, respectively) confirmed the appropriate sensitivity of the assay to changes in β-oxidation. The lipid β-oxidation assay was also performed for three pairs of replicon cell lines and the corresponding IFN-cured cell lines. In both subgenomic-replicon (SGR) cell lines for genotype 2a (Y-19) and genotype 1b (R6 FLR-N), SGR RNA replication caused impairment of lipid β-oxidation by approximately 24.5% and 35.6% (compared to lipid β-oxidation in the cured cell lines), respectively. A full-length genomic replicon of genotype 2a (K-2) inhibited lipid β-oxidation more dramatically (46.0%) than in the cured cell control, implying that the additionally expressed viral proteins (core, E1, E2, p7, and NS2) might have caused additional inhibition of lipid β-oxidation. Although lipid β-oxidation was significantly impaired, it was not associated with a loss in cell viability, as confirmed by using a Cell Counting Kit-8 (CCK-8; Dojindo, Japan) assay (Fig. 1D and F).

**FIG 2:**
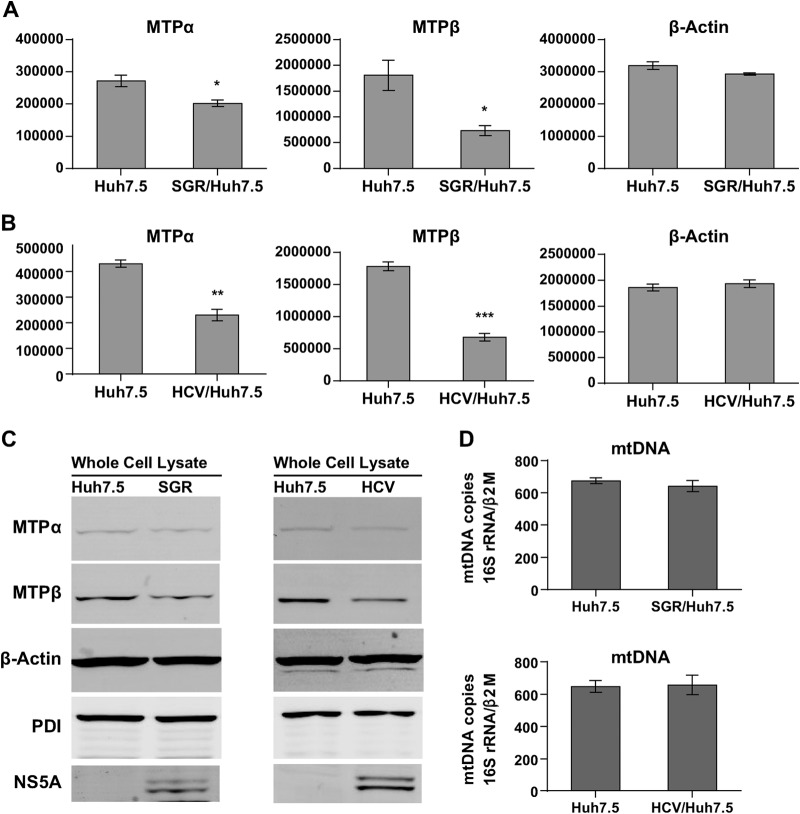
HCV replication causes a decrease in MTPα and -β protein expression. (A to C) Whole-cell lysates from naive Huh7.5 cells, SGR-JFH-1-harboring cells, or HCV-infected cells (Jc1 MOI of 0.05 FFU/cell, day 7 postinfection) were analyzed by Western blotting by using conjugated infrared secondary antibodies. (A and B) Quantitative measurements of band intensities were taken using a LI-COR Odyssey Sa infrared imaging system and calculated by image studio software (*n* = 4). The *y*-axis scales are arbitrary units, representing protein band intensities. Asterisks indicate *P* values as follows: *, <0.05; **, <0.001; and ***, <0.0001. (C) Whole-cell lysates were analyzed to compare levels of expression of MTPα, MTPβ, and cellular markers. Lysates (3 μg protein) were analyzed by infrared Western blotting. β-Actin is an internal loading control. PDI is an endoplasmic reticulum- and mitochondrion-associated membrane protein. (D) mtDNA quantitations were performed for Huh7.5 SGR-harboring cells (upper panel) or HCV-infected cells (lower panel).

To investigate the mechanism of viral intervention with lipid β-oxidation, we measured the protein expression levels of MTPα and MTPβ. Whole-cell lysates from Huh7.5, SGR-harboring, or HCV-infected cells were analyzed by quantitative infrared Western blotting. The results show that both SGR replication and HCV infection downregulated levels of both MTPα and MTPβ (Fig. 2A to C) compared to levels in the β-actin and PDI controls. In the case of HCV-infected Huh7.5 cells, levels of MTPα and -β proteins were reduced by 46.6% and 61.9%, respectively (Fig. 2B). Considering that ablation of MTPα in mice caused embryonic lethality, a 50% reduction of MTP protein expression and enzyme activity may cause severe outcomes in terms of cellular physiology and pathology (35).

A recent study revealed that HCV infection promotes mitophagy, a selective degradation of mitochondria by autophagy (34). In light of this finding, we speculated that HCV infection might cause a decline in the numbers of mitochondria per cell; however, quantitative PCR for mitochondrial DNA (16S RNA gene borne in mtDNA) revealed that neither HCV infection nor SGR replication caused a change in the levels of mtDNA (Fig. 2D). This implies that attenuation of lipid β-oxidation was not caused by a decrease in the mitochondrial content within the cells. Although little is known about how mitochondrial biogenesis can be affected by HCV infection, HCV-induced mitophagy might be compensated for by upregulation of mitochondrial biogenesis to maintain the overall mitochondrial number. To further investigate how MTP gene expression may be affected in the course of HCV infection, we performed a series of quantitative, real-time reverse transcription (RT)-PCR experiments.

### HCV attenuates MTP expression at the transcriptional level.

**FIG 3:**
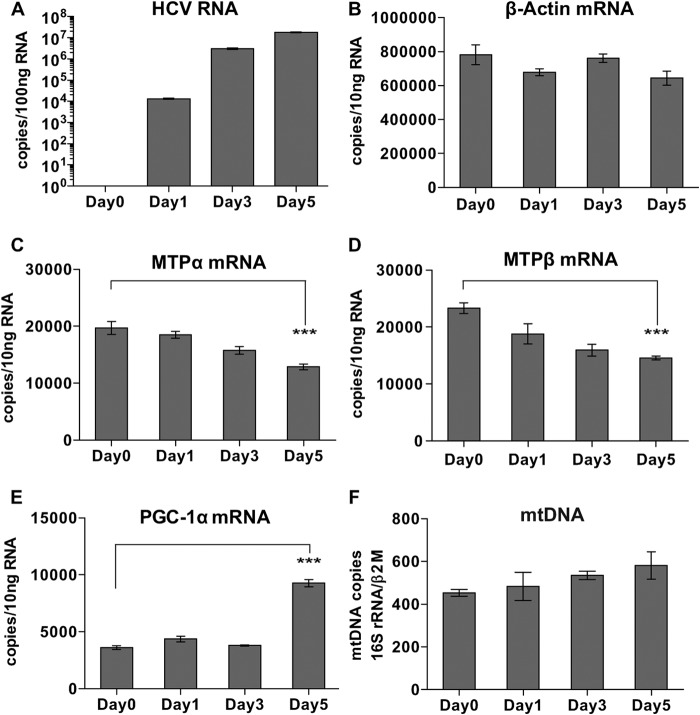
HCV attenuates MTP protein expression at the transcriptional level. The abundances of HCV RNA (A), β-actin mRNA (B), MTPα (C), MTPβ (D), and PGC-1α mRNA (E) were examined by real-time RT-PCR in HCV-infected cells. ***, *P* < 0.001. (F) mtDNA was quantitated by real-time PCR.

To further investigate the mechanism of the HCV effect on lipid β-oxidation, MTPα and MTPβ mRNAs were quantified by quantitative RT-PCR. Huh7.5 cells were infected with HCV (JFH-1, MOI = 0.05), and then mRNA levels for MTPα, MTPβ, and β-actin, together with levels of HCV RNA, were monitored for 5 days. By day 5 postinfection, MTPα and MTPβ mRNAs were downregulated by approximately 35% and 38%, respectively (Fig. 3C and D, respectively), while HCV RNA reached a plateau at day 5 (Fig. 3A). β-Actin mRNA levels were unaffected throughout this period (Fig. 3B). Transcription of MTPα and MTPβ was inhibited in parallel. This may be because these two genes share a promoter region; both the MTPα and MTPβ genes are located on chromosome 2p23, where they are transcribed in opposite directions from a shared bidirectional promoter of 350 bases (36). It is also noteworthy that the level of transcriptional attenuation was comparable to those of protein and enzymatic downregulation, as described above. These data demonstrate that HCV regulation of MTP expression most likely occurs at the transcriptional level. We also assessed the level of peroxisome proliferator-activated receptor γ coactivator 1α (PGC-1α) mRNA as a marker of mitochondrial biogenesis (37). Interestingly, the level of PGC-1α was elevated at day 5 after HCV infection (Fig. 3E). This implies that as well as reducing the transcription of MTPα and MTPβ, HCV increased mitochondrial biogenesis, perhaps to compensate for the loss of MTP. Consistently with this, there was no reduction in the levels of mtDNA (and therefore mitochondrial numbers) over the period of HCV infection.

### Inhibition of MTP expression impairs overall lipid β-oxidation activity.

**FIG 4:**
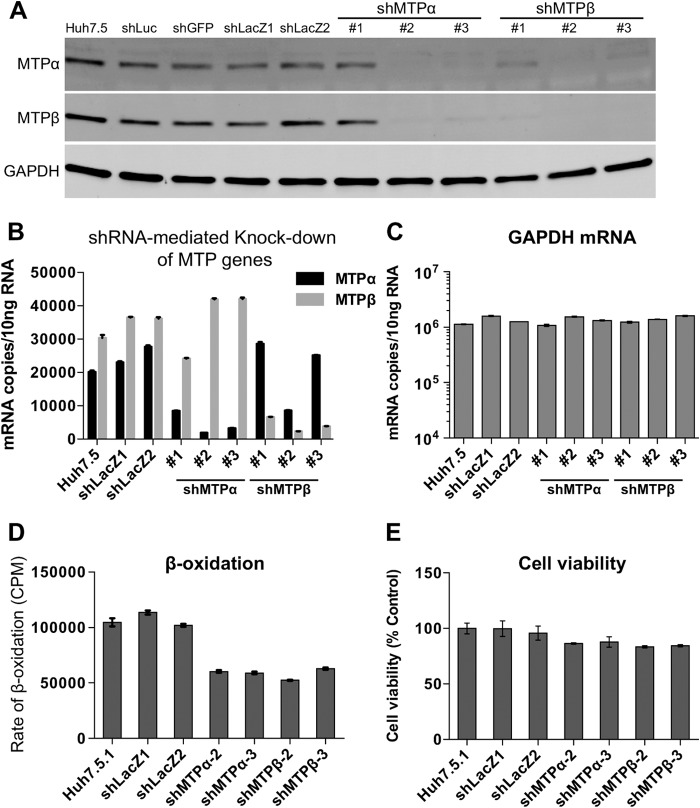
shRNA-mediated knockdown of MTPα and MTPβ. Lentiviral-vector-mediated shRNAs as indicated were used to downregulate gene expression. Huh7.5 cells were transduced with the indicated lentiviral shRNA vectors. After 2 days of incubation, efficient gene transduction was confirmed by fluorescence microscopy using GFP as a marker. Cells were harvested at day 4 after lentiviral infection. (A) Western blot analysis for expression of MTPα, MTPβ, and GAPDH. Quantitative RT-PCR analysis of MTPα and MTPβ (B) and GAPDH (C) mRNA levels. (D) Huh7.5 cells were fed with [^3^H]palmitate, and ^3^H_2_O was assayed as a measure of lipid β-oxidation. (E) Cell viability was measured by the WST-8 assay.

In order to study how MTP function may relate to HCV replication, we constructed lentiviral vectors expressing shRNA targeted to either MTPα or MTPβ, along with control lentiviruses targeting luciferase (Luc), GFP, and β-galactosidase (LacZ). Three lentiviruses each for MTPα and MTPβ were constructed, and 5 of these 6 shMTPs effectively downregulated protein expression. The exception was shMTPα number 1 (Fig. 4A), although this shRNA did achieve a 50% knockdown of mRNA levels (Fig. 4B). RNA interference triggered coincident ablation of both MTPα and MTPβ, and where shRNA mediated ablation of either subunit, it also ablated the other. This was expected because, as mentioned above, transcription of MTPα and MTPβ mRNAs are tightly correlated, a relationship that seems to be extended to posttranscriptional processing and translation, until they form a mature octamer complex. At the level of mRNA expression, each shMTP construct specifically downregulated its cognate target mRNA, except for shMTPβ number 2, which also caused inhibition of MTPα transcription (Fig. 4B). None of the shRNAs tested in this study affected GAPDH mRNA levels (Fig. 4C) or GAPDH expression (Fig. 4A). Furthermore, we confirmed that the shRNA-mediated knockdown of MTP genes led to the impairment of overall lipid β-oxidation activity (Fig. 4D), without affecting their viabilities (Fig. 4E). We proceeded to test MTP-depleted cells for effects on HCV replication.

**FIG 5:**
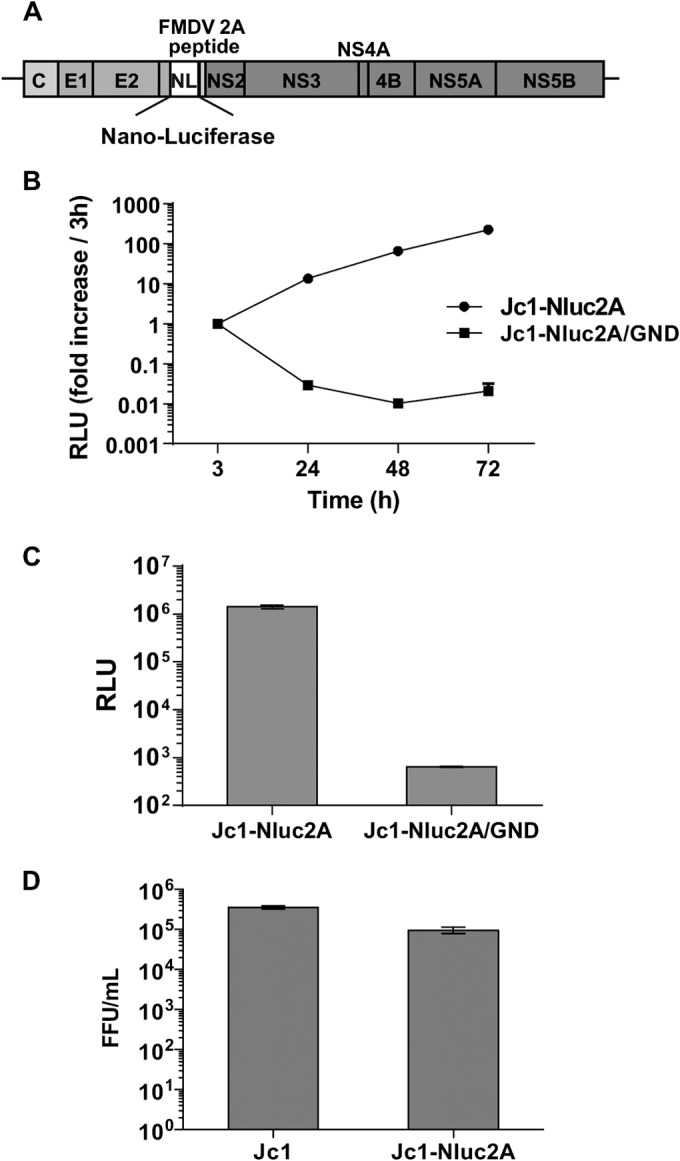
Characterization of the NanoLuc reporter virus (Jc1-p7NLuc2A). (A) Schematic illustration of Jc1-p7NLuc2A. Nanoluciferase is the smallest luciferase reporter gene; thus, this construct is the shortest monocistronic, full-genomic reporter HCV construct. (B) The replication kinetics of Jc1-p7NLuc2A was evaluated by the nanoluciferase Glo assay following RNA electroporation into Huh7.5 cells. The GND derivative is a polymerase-inactive negative control, unable to replicate. (C) Cultured supernatants collected from day 3 of the experiment shown in panel B were overlaid onto 10^4^ cells in 96-well plates. After 2 days of incubation, infected cells were tested by the Nano-Glo luciferase assay to confirm reporter gene transduction via virus infection. (D) Comparison of the infectivities of Jc1-p7NLuc2A and the parental Jc1 virus following electroporation of the corresponding RNAs into Huh7.5 cells.

**FIG 6:**
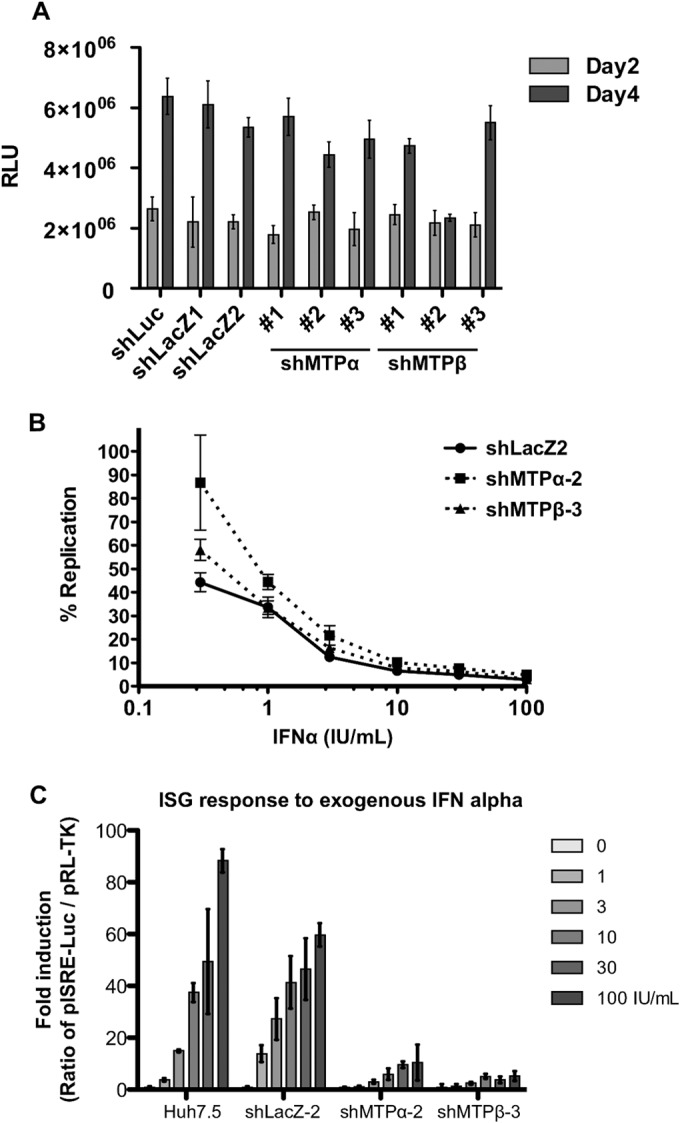
MTP protein depletion leads to suppression of the type I IFN response. MTP depletion does not directly affect HCV replication but suppresses the type I IFN response. (A) Huh7.5 cells were transduced with the indicated lentiviral shRNA vectors, and after 24 h of incubation, cells were infected with HCV Jc1-p7NLuc2A (MOI = 0.05 FFU/cell) (Fig. 5). Infected cells were lysed at days 2 and 4 after HCV infection and assayed by the nanoluciferase A Glo assay to assess HCV replication. (B) MTP depletion renders host cells less responsive to exogenous IFN-α to suppress HCV replication. Huh7.5 cells were transduced with the indicated lentiviral shRNA vectors. Two days later, cells were subsequently infected with HCV (MOI = 0.05 FFU/cell), incubated for 4 days, and plated in a 96-well plate at 1 × 10^4^ cells per well for IFN-α treatment for a further 2 days. The levels of viral replication were assayed using Nano-Glo luciferase assay reagent as per the manufacturer's instruction (*n* = 6). (C) MTP depletion reduces the ISG response. Lentiviral-vector-mediated shRNAs as indicated were used to downregulate MTP gene expression. Transduced cells were transfected with pISRE-Luc and pRL-TK and then stimulated with IFN-α at the indicated concentrations for 5 h prior to analysis by the dual-luciferase reporter assay (*n* = 4). Error bars represent SEM.

A reporter virus construct carrying a novel luciferase gene (NanoLuc) was generated for this experiment (Fig. 5A). This reporter virus construct, designated Jc1-p7NLuc2A, was constructed in a fashion analogous to that used to construct p7-Rluc2A (described in reference 38), and it replicated efficiently in Huh7.5 cells upon RNA transfection (Fig. 5B). Reporter virus particles were very effectively produced by transfecting RNA into Huh7.5 cells (Fig. 5C and D) and used to infect MTP-depleted cells; however, the results showed that MTP-depleted cells supported HCV replication as well as control cells did (Fig. 6A), except with shMTPβ number 2, which reduced HCV replication at a late time point (day 4). Interestingly, this shRNA also reduced MTPα transcription (Fig. 4B), suggesting that depletion of both MTP subunits was detrimental to HCV replication or that this shRNA might have an off-target effect. For this reason, shMTPβ number 2 was not used in the subsequent IFN experiments. These data are consistent with the fact that HCV infection itself downregulates MTP protein expression and lipid β-oxidation so that the effect of shRNA-mediated knockdown can be overshadowed by this autonomous reduction in MTPs by HCV. We also constructed lentivirus vectors to overexpress either MTPα, MTPβ, or both MTP subunits. However, although transduction of Huh7.5 cells with these vectors was able to increase levels of MTP expression, there was no effect on levels of lipid β-oxidation (data not shown), suggesting that other enzymes in the pathway were rate limiting or, alternatively, that merely overexpressing MTP does not result in an increase in the transport of these proteins to the mitochondria and the assembly of a functional enzymatic complex.

### MTP protein depletion leads to deregulated responses to IFN-α.

HCV has been shown to alter the functions of mitochondria (39). Mitochondria not only are important for energy production but also function in the innate immune response against intracellular microbe infection, including viruses (40). HCV has a specific strategy to evade this function, as the protease NS3 cleaves the mitochondrial antiviral signaling protein (MAVS) to prevent the association of this adaptor with the mitochondrial membrane, thereby blocking activation of downstream effectors (e.g., NF-κB and IRF3). This effect is important in the determination of host cell permissiveness to HCV infection. In light of these facts, we speculated that impaired mitochondrial lipid β-oxidation might have an effect on the interferon response pathway. First, we infected MTP-depleted cells with the NanoLuc reporter virus and tested the ability of exogenously added interferon to control HCV infection. In comparison with the control cell line transduced with the LacZ targeting lentivirus (shLacZ), MTP-depleted cells were less able to suppress viral replication in response to exogenously added IFN-α (Fig. 6B). The lack of responsiveness to IFN-α was more evident when cells were treated with lower doses of IFN-α (from 0.3 to 3 IU/ml). At higher concentrations, this effect was less evident. Paralleling the lack of effect of MTP overexpression on lipid β-oxidation, transduction with lentivirus vectors expressing either MTPα, MTPβ, or both MTP subunits had no significant effect on IFN-α responsiveness (data not shown).

To investigate the lack of IFN responsiveness following shRNA ablation of MTP expression, we analyzed transcription of interferon-stimulated genes (ISG) following exogenous addition of IFN-α. We used a reporter construct (pISRE-Luc) in which luciferase expression was controlled by the interferon-stimulated response element (ISRE). As shown in Fig. 6C, ISRE responses to IFN-α were dramatically suppressed when MTP expression was ablated by shRNA. These data help to explain why MTP depletion may cause a lack of responsiveness to exogenous IFN-α. However, it is noteworthy that the effect on ISRE transcription was seen at all IFN concentrations, suggesting that there must be other effects of MTP depletion.

## DISCUSSION

Mitochondrial lipid β-oxidation is a major bioenergetic pathway within the liver for the production of ATP from fatty acids. Metabolic profiling studies have revealed that the cellular ATP concentration is significantly lowered by HCV infection (32, 41), and HCV infection alters the host energy expenditure profile in many ways (20–22). HCV is known to interact with mitochondria to induce reactive oxygen production, which is also thought to lead to impairment of lipid β-oxidation (39). Furthermore, a recent clinical study showed that under fasting conditions, the total ketone body concentration was significantly lower for chronic HCV patients than for their healthy uninfected counterparts (19). This indicates that mitochondrial lipid β-oxidation is impaired in the livers of HCV-infected patients, because ketogenesis is a liver-specific metabolism that occurs in mitochondria and is directly coupled to mitochondrial lipid β-oxidation. Combining these data with the data in this study, it is very likely that HCV suppresses mitochondrial lipid β-oxidation to alter cellular bioenergetics. This situation may be specific to HCV as, in contrast to our observations, the related Dengue virus (also a member of the Flaviviridae) has been shown to upregulate β-oxidation to facilitate lipid consumption and ATP production by infected cells (29). The phenotype of impaired mitochondrial lipid β-oxidation has been well studied and documented in the case of MTP gene deficiency. Genetic defects in mitochondrial lipid β-oxidation are recessively inherited and cause pediatric and maternal morbidity and mortality. Children with this disorder suffer nonketotic hypoglycemia, steatohepatitis, and skeletal and cardiac myopathy (42). Consistently with this phenotype in humans, MTPα knockout mice are embryonic lethal (35), and heterozygous mice develop hepatic steatosis (24). HCV infection attenuated lipid β-oxidation by more than 50% (Fig. 1), and such a degree of β-oxidation inhibition may severely impact the overall health of the host, providing clues as to the molecular mechanisms underpinning the pathogenesis of HCV infection.

The mitochondrial lipid β-oxidation pathway comprises mitochondrial trifunctional proteins and other enzymes, which include very-long-, long-, medium-, and short-chain acyl-CoA dehydrogenases (CADs). Interestingly, a recent report showed that medium and short-chain CAD expression are also suppressed by HCV (27). Phenotypes of these gene defects are marked with intolerance to cold and fasting and an inability to convert fatty acids to energy (ATP). HCV-induced impairment of lipid β-oxidation can cause the accumulation of lipids inside liver cells, leading to steatosis, although it is not yet fully confirmed to what degree this can cause severe fatty liver, along with increased lipogenesis and impaired VLDL secretion.

To discuss possible mechanisms for how HCV attenuates mitochondrial lipid β-oxidation, we need to consider multiple factors. In this study, we observed that HCV attenuates the transcription of both MTP genes. Data obtained by an MTP promoter/reporter assay revealed that inflammatory cytokines, such as tumor necrosis factor alpha (TNF-α), interleukin 1β (IL-1β), and IFN-α inhibited the transcription of both MTP genes (data not shown). HCV infection is capable of inducing the expression of these cytokines, and this may therefore drive a negative-feedback loop for the suppression of lipid β-oxidation. To the best of our knowledge, Sp1 is the only transcription factor which has been identified to bind to the MTP promoter region (36). Further studies are therefore needed to reveal how MTP promoter activity might be modulated by HCV infection. Intriguingly, we also observed a physical interaction between HCV NS5A and both MTPα and MTPβ (data not shown); indeed, this was the observation that first drew our attention to the β-oxidation pathway. It is possible therefore that HCV has an impact on β-oxidation at multiple levels, and it would be intriguing to determine whether NS5A in some way modulates the activity of the MTP complex.

In the last section of this study, we described that inhibition of mitochondrial lipid β-oxidation correlated with a concomitant reduction in the transcriptional response to IFN-α (Fig. 6C). This is perhaps unsurprising, as mitochondria orchestrate a diverse cellular response in survival situations (reviewed in reference 43), and indeed cell-fate-decisive signaling cascades take place on the surfaces of mitochondria. Although the mechanism by which β-oxidation may be linked to IFN responsiveness remains obscure, a number of other observations may provide some clues: HCV protein expression (of either core, NS3, or NS5A) can suppress type I IFN responses by disrupting STAT1 phosphorylation and activation (44–46). Phosphorylation activation of STAT1 and a subsequent nuclear location of p-STAT1 are required for ISRE-mediated gene transcription. Interestingly, core, NS3, and NS5A are all known to interact with mitochondria, and recent studies suggest that some STATs also potentially localize to mitochondria (47). It will be interesting to see how localization and activation of STATs may be affected by HCV infection-mediated attenuation of lipid β-oxidation or when MTP proteins are depleted by shRNA.

In summary, it is apparent that HCV interacts with mitochondria in multiple ways to modulate host energy metabolism and antiviral defense. Our evidence points to a link between mitochondrial bioenergetics and the type I IFN response. Further study of this aspect of the host-virus interaction will lead to a better understanding of HCV biology and how current standard therapy can be helped by regaining mitochondrial functions.
